# Revision arthroplasty versus open reduction and internal fixation in Vancouver type B2 and B3 periprosthetic femoral fractures: results from a matched pairs analysis of the registry for geriatric trauma of the DGU^®^

**DOI:** 10.1007/s00402-025-06126-x

**Published:** 2025-11-25

**Authors:** Anna Schweer, Hannah Schmidt, Bastian Pass, Carsten Schoeneberg, Rene Aigner, Rene Burchard, Artur Barsumyan, Christopher Bliemel, on behalf ot the AltersTraumaRegister DGU

**Affiliations:** 1 Center for Trauma Surgery and Orthopedics, Lahn-Dill-Kliniken Wetzlar, Wetzlar, Germany; 2 Academy of Trauma Surgery (AUC) GmbH, Munich, Germany; 3 Philipps-Universität Marburg, Marburg, Germany; 4 Department of Orthopedic and Emergency Surgery, Alfried Krupp Hospital, Essen, Germany; 5 Center for Orthopedics and Trauma Surgery, University Hospital of Giessen and Marburg, Marburg, Germany; 6 Department of Orthopedics and Trauma Surgery, Lahn-Dill-Kliniken Dillenburg, Dillenburg, Germany; 7 Center for Trauma Surgery and Orthopedics, Lahn-Dill-Kliniken Wetzlar, Wetzlar, Germany

**Keywords:** Periprosthetic femoral fracture, Vancouver type B2 and B3 fractures, Mortality, Quality of life, Revision arthroplasty, Open reduction internal fixation, AltersTraumaRegister DGU

## Abstract

**Background and objectives:**

Owing to a lack of evidence, the appropriate surgical treatment strategy for geriatric patients with Vancouver type B2 or B3 periprosthetic femoral fractures (PFFs) remains unclear. Data from a large international geriatric trauma registry were analyzed to investigate the medical care situation of such patients, as well as to examine the outcomes related to revision arthroplasty (RA) or open reduction and internal fixation (ORIF).

**Materials and methods:**

Datasets from the Registry for Geriatric Trauma of the German Trauma Society (Deutsche Gesellschaft für Unfallchirurgie [DGU]) (ATR-DGU) were analyzed. The ATR-DGU is a prospective, multicenter registry that provides information on geriatric trauma patients. All patients who underwent surgery for PFF were included in this analysis. The outcome parameters included the mortality rate during hospitalization and at the 120-day follow-up, as well as mobility, the EQ-5D-5 L score and the reoperation rate, and were analyzed in relation to RA versus ORIF in Vancouver type B2 or B3 PFF patients.

**Results:**

A total of 607 patients with Vancouver type B2 or B3 PFF met the inclusion criteria. Among these patients, 420 underwent RA, and ORIF was performed in 187 patients. Regression analysis of the parameters collected during the acute phase revealed that after 2:1 matching, compared with the RA group, the ORIF group had significantly lower odds for full weight bearing allowed one day after surgery (OR: 0.49; *p* < 0.001); walking ability after seven days (OR: 0.56; *p* = 0.005); and the occurrence of nonsurgical complications (OR: 0.59; *p* = 0.012). The probability of death during follow-up and the EQ-5D-5 L score after seven and 120 days remained unaffected.

**Conclusions:**

The results of the present study support the estimate that ORIF represents a valid treatment alternative for Vancouver type B2 and B3 PFFs, as comparable midterm outcomes were demonstrated for each patient group. However, individualized decisions should always be made, especially for multimorbid geriatric patients, to reduce complications.

## Introduction

Owing to the aging population with increased activity levels, particularly in western industrialized countries, a significant increase in primary hip arthroplasty has occurred [1–3]. High rates of hip arthroplasty inevitably lead to increased rates of periprosthetic femoral fracture (PFF) [4, 5]. Today, with a rate of 15.9%, PFF represents the third most common reason for revision surgery according to the German arthroplasty registry (Endoprothesenregister Deutschland (EPRD)) 2024 annual report [6].

The Swedish registry data revealed that 3.5% of patients who underwent total hip arthroplasty underwent PFF within the first 20 years following implantation. Additionally, for the United States of America (USA), an increase of up to 4.6% every ten years is predicted within the next 30 years [5, 7].

According to the Vancouver classification established by *Duncan and Masri* [8], approximately 34% of all PFFs are Vancouver type B2 (24.5%) and B3 (9.2%) fractures, which is also reflected by the Swedish arthroplasty registry [9, 10].

Revision arthroplasty (RA) is considered to be the gold standard and has therefore been recommended for Vancouver type B2 and B3 PFFs for a long time [11–13]. Nevertheless, RA represents a major surgical procedure with increased perioperative complications, especially in multimorbid patients with a poor overall constitution [14, 15]. According to previous studies, the mortality rates for RA due to PFF are high, ranging from 9.4 to 13.4% ^10,15,16^. Even though patients’ postoperative autonomy and quality of life have reached the focus of interest in recent years [17], owing to the complications mentioned above, RA is not a suitable option for every geriatric patient.

In recent years, a paradigm shift has occurred, indicating that open reduction and internal fixation (ORIF) for Vancouver type B2 and B3 PFFs might represent an adequate alternative, especially in multimorbid elderly patients. Recent studies have shown that ORIF provides comparable results in terms of postoperative mobility, as well as revision, complication and mortality rates [18–20].

To shed further light on this controversial point of debate and clarify the reality of surgical care for Vancouver type B2 and B3 PFFs, we analyzed data from the Registry for Geriatric Trauma (AltersTraumaRegister DGU^®^, ATR-DGU) of the German Trauma Society (Deutsche Gesellschaft für Unfallchirurgie (DGU)). It was hypothesized that compared with ORIF, RA for Vancouver type B2 or B3 PFF would result in better postoperative mobility with higher perioperative mortality rates.

## Materials and methods

### Data source

The DGU established the ATR-DGU in 2016 to enable pseudonymized and standardized documentation of geriatric patients with proximal femur fractures. The ATR-DGU collects data across six consecutive time points: admission, preoperative phase, operative phase, the first postoperative week, discharge or transfer and an optional follow-up at 120 days postoperatively. The follow-up examination includes assessments of walking ability, additional surgeries and the health-related quality of life questionnaire European Quality of Life 5 Dimensions 5 Level Version (EQ-5D-5 L) on postoperative Day 120. Data documentation, management and analysis are coordinated by the AUC -Academy for Trauma Surgery (Akademie der Unfallchirurgie GmbH, AUC), while scientific oversight is provided by the Working Committee on Geriatric Trauma Registry DGU^®^. Data are submitted pseudonymized via a web-based application into a central database, and scientific analyses require approval through a peer review process based on publication guidelines set by the Working Committee. Currently, hospitals from Germany, Austria and Switzerland participate in the ATR-DGU, contributing approximately 16,300 cases from 153 hospitals annually. Certified centers for geriatric trauma (AltersTraumaZentrum DGU^®^) are mandated to participate in the ATR-DGU. The present study is in accordance with the publication guidelines of the ATR-DGU and is registered as ATR-DGU project ID 2023-003.

### Patients

The inclusion criteria of the ATR-DGU are hip fractures, including periprosthetic and peri-implant fractures, with an indication for surgery in patients aged 70 years or older. Since Vancouver type B2 and B3 fractures have been recorded only since 2022, we included patients from 2022 onward in this study. Patients without Vancouver type B2 or B3 fractures and those with pathological fractures were excluded.

### Covariates

The following covariates were measured: age, sex, American Society of Anesthesiologists (ASA) score (1–5), Clinical Frailty Scale (CFS), Identification of Seniors At Risk (ISAR) score, anticoagulants, concomitant injuries, prefracture residential status, prefracture walking ability, and screening for delirium.

### Outcomes

Mortality during the acute hospital stay and until the 120-day follow-up, walking ability seven days after surgery, reoperation during acute hospitalization, full weight bearing allowed after surgery, nonsurgical complications during acute hospitalization, and the EQ-5D-5 L score at seven and 120 days after surgery were recorded as outcome parameters.

### Statistical analysis

All the statistical analyses were performed using the statistical software R version 4.1.2 (Foundation for Statistical Computing, Vienna, Austria). Descriptive analyses summarized categorical variables as absolute and relative frequencies, while continuous variables are reported as medians with interquartile ranges (IQRs). Analyses accounted for missing data by including only patients with complete records for the respective parameter.

The EQ-5D-5 L questionnaire results were converted into a single index value ranging from − 0.661 (worst health status) to 1 (best health status) using the German Ludwig value set (Version 2.1) ^21^.

Group comparisons (RA vs. ORIF) were conducted using Pearson’s chi-square test or Fisher’s exact test for categorical variables and the Wilcoxon signed-rank test for continuous variables.

To control for differences between the demographics of the two groups, matching was performed on all complete cases using the MatchIt package [22]. Sex, age, ASA score and prefracture walking ability were considered matching variables. A good balance was achieved with 2:1 nearest neighbor matching and the difference in the propensity score as the distance measure. The standardized mean difference for all the matching variables was < 0.1.

Linear and logistic regression models were used to assess the effects of the treatment methods on a range of outcomes. Postoperative living situations were additionally adjusted for their preoperative level. The results are presented as regression coefficients (β) for linear regression models and odds ratios (OR) for logistic regression models, with 95% confidence intervals (CI). A significance level of *p* < 0.05 was applied throughout.

## Results

### Acute care data

A total of 607 patients from 114 geriatric trauma centers (recruitment period 2022–2023) with Vancouver type B2 or B3 PFF met the inclusion criteria. Among these patients, 420 (69.2%) underwent implant replacement, and in 187 patients (30.8%), fracture fixation with osteosynthesis was performed (Table 1).

**Table 1:** Data on patients with periprosthetic femoral fractures type Vancouver B2/B3

Parameter	All patients (before matching)			After matching		
	Revision arthroplasty (RA)	Osteosynthesis (ORIF)	*p*-value	Revision arthroplasty (RA)	Osteosynthesis (ORIF)	*p*-value
Number of patients	420 (69,2%)	187 (30,8%)		326 (66,7%)	163 (33,3%)	
Gender**						
Male	148 (35,2%)	50 (26,7%)	0,049	99 (30,4%)	43 (26,4%)	0,418
Female	272 (64,8%)	137 (73,3%)		227 (69,6%)	120 (73,6%)	
Patient age (year) Median [IQR] *						
Missings	8 84 [80, 88]	3 86 [81, 89]	0,034	85 [80, 88]	85 [81, 89]	0,266
ASA score*						
Missings	7	9	0,687	0	0	0,832
1	3 (0,7%)	5 (2,8%)		1 (0,3%)	4 (2,5%)	
2	77 (18,6%)	32 (18,0%)		62 (19,0%)	28 (17,2%)	
3	296 (71,7%)	125 (70,2%)		232 (71,2%)	116 (71,2%)	
4 and 5	37 (9,0%)	16 (9,0%)		31 (9,5%)	15 (9,2%)	
CFS score*						
Missings	149	67	0,795	95	50	0,908
1	4 (1,5%)	5 (4,2%)		3 (1,3%)	4 (3,5%)	
2	28 (10,3%)	8 (6,7%)		22 (9,5%)	7 (6,2%)	
3	43 (15,9%)	20 (16,7%)		34 (14,7%)	19 (16,8%)	
4	45 (16,6%)	21 (17,5%)		40 (17,3%)	20 (17,7%)	
5	43 (15,9%)	14 (11,7%)		36 (15,6%)	12 (10,6%)	
6	59 (21,8%)	28 (23,3%)		51 (22,1%)	28 (24,8%)	
7	40 (14,8%)	21 (17,5%)		37 (16,0%)	20 (17,7%)	
8	9 (3,3%)	3 (2,5%)		8 (3,5%)	3 (2,7%)	
ISAR score*						
Missings	113	65	0,035	77	52	0,062
0	19 (6,2%)	6 (4,9%)		13 (5,2%)	5 (4,5%)	
1	42 (13,7%)	13 (10,7%)		34 (13,7%)	12 (10,8%)	
2	76 (24,8%)	23 (18,9%)		60 (24,1%)	22 (19,8%)	
3	87 (28,3%)	35 (28,7%)		76 (30,5%)	30 (27,0%)	
4	58 (18,9%)	32 (26,2%)		44 (17,7%)	29 (26,1%)	
5	21 (6,8%)	10 (8,2%)		18 (7,2%)	10 (9,0%)	
6	4 (1,3%)	3 (2,5%)		4 (1,6%)	3 (2,7%)	
Anticoagulatory drugs**						
Missings (before matching 14/after matching 9)			0,707			0,836
Yes	251 (60,5%)	104 (58,4%)		194 (60,1%)	92 (58,6%)	
No	164 (39,5%)	74 (41,6%)		129 (39,9%)	65 (41,4%)	
Cocomitant injuries**						
Missings (before matching 7/after matching 5)			0,524			0,614
Yes	40 (9,6%)	14 (7,6%)		30 (9,3%)	12 (7,5%)	
No	376 (90,4%)	170 (92,4%)		293 (90,7%)	149 (92,6%)	
Prefracture residental status***						
Missings (before matching 6/after matching 2)			0,097			0,051
At home	365 (88,2%)	153 (81,8%)		287 (88,6%)	132 (81,0%)	
Nursing home	46 (11,1%)	32 (17,1%)		35 (10,8%)	29 (17,8%)	
Other	3 (0,7%)	2 (1,1%)		2 (0,6%)	2 (1,2%)	
Prefracture walking ability**						
Missings (before matching 55/after matching 0)			0,391			0,9
Independent without walking aids	136 (35,9%)	49 (28,3%)		100 (30,7%)	45 (27,6%)	
Ability to walk inside	44 (11,6%)	21 (12,1%)		43 (13,2%)	21 (12,9%)	
Ability to walk with walking stick or crutch	57 (15,0%)	25 (14,5%)		48 (14,7%)	22 (13,5%)	
Ability to walk with two crutches or a walker	126 (33,3%)	71 (41,0%)		121 (37,1%)	68 (41,7%)	
No functional walking ability	16 (4,2%)	7 (4,1%)		14 (4,3%)	7 (4,3%)	
Screening for delirium**						
Missings (before matching 175/after matching128)			1,000			0,959
Positive	59 (18,9%)	22 (18,3%)		48 (18,8%)	19 (17,9%)	
Negative	253 (81,1%)	98 (81,7%)		207 (81,2%)	87 (82,1%)	
Death during stay in the acute hospital **						
Missings (before matching 3/after matching 0)			0,216			0,379
Yes	28 (6,7%)	7 (3,8%)		22 (6,8%)	7 (4,3%)	
No	390 (93,3%)	179 (96,2%)		304 (93,3%)	156 (95,7%)	
Reoperation within initial acute hospital stay**						
Missings (before matching 33/after matching 26)			0,162			0,089
Yes	36 (9%)	9 (5,2%)		33 (10,6%)	8 (5,3%)	
No	364 (91%)	165 (94,8%)		279 (89,4%)	143 (94,7%)	
Ability to walk at the 7th postoperative day**						
Missings (before matching 40/after matching 25)			0,015			0,046
With walking buck	52 (13,3%)	17 (9,7%)		41 (13,3%)	17 (11,0%)	
With a walker	125 (32,0%)	51 (29,0%)		100 (32,4%)	46 (29,7%)	
With a rollator	62 (15,9%)	21 (11,9%)		53 (17,2%)	18 (11,6%)	
With walking crutch	30 (7,7%)	7 (4,0%)		19 (6,2%)	5 (3,2%)	
Not possible	122 (31,2%)	80 (45,5%)		96 (31,1%)	69 (44,5%)	
Full weight bearing allowed**						
Missings (before matching 11/after matching 8)			< 0,001			< 0,001
Yes	226 (54,9%)	68 (37,0%)		175 (54,5%)	59 (36,9%)	
No	186 (45,2%)	116 (63,0%)		146 (45,5%)	101 (63,1%)	
Other complications (multiple answers possible) **						
Missings (before matching 15/after matching 13)			0,003			0,023
Total	192 (46,8%)	61 (33,5%)		149 (46,9%)	56 (35,4%)	
No	218 (53,2%)	121 (66,5%)		169 (53,1%)	102 (64,6%)	
Decubitus	23 (5,61%)	2 (1,1%)		15 (4,7%)	1 (0,6%)	
Cardiac infarction	1 (0,2%)	3 (1,7%)		1 (0,3%)	3 (1,9%)	
Thrombosis	5 (1,2%)	1 (0,6%)		3 (0,9%)	1 (0,6%)	
Pulmonary embolism	5 (1,2%)	1 (0,6%)		5 (1,6%)	1 (0,6%)	
Renal insufficiency	36 (8,8%)	10 (5,5%)		29 (9,1%)	10 (6,3%)	
Delirium	57 (13,9%)	9 (5,0%)		48 (15,1%)	9 (5,7%)	
Cystitis	51 (12,4%)	24 (13,2%)		38 (12,0%)	22 (13,9%)	
Pneumonia	36 (8,8%)	10 (5,5%)		26 (8,2%)	9 (5,7%)	
Other	97 (23,7%)	27 (14,8%)		79 (24,8%)	26 (16,5%)	
Discharge from hospital**						
Rehabilitation-clinic	15 (3,9%)	3 (1,7%)	0,103	12 (4,0%)	2 (1,3%)	0,038
Acute geriatrics	73 (18,7%)	38 (21,2%)		49 (16,1%)	33 (21,2%)	
Other hospital	22 (5,6%)	13 (7,3%)		19 (6,3%)	13 (8,3%)	
Geriatric rehabilitation-clinic	77 (19,7%)	21 (11,7%)		64 (21,1%)	18 (11,5%)	
Nursing home	111 (28,5%)	63 (35,2%)		90 (29,6%)	57 (36,5%)	
At home	92 (23,6%)	41 (22,9%)		70 (23,0%)	33 (21,2%)	
EQ-5D-5 L Index Median [IQR] *	0,405 [0,198, 0,662]	0,402 [0,220, 0,662]	0,965	0,401 [0,186, 0,662]	0,401 [0,201, 0,642]	0,866

* Wilcoxon-sign-rank test ** Pearson-Chi-square test. *** Exact test according to Fisher IQR: interquartile range

Gender analysis revealed that significantly more women than men were affected by Vancouver type B2 or B3 PFF. In the RA group, 64.8% (*n* = 272) and in the ORIF group, 73.3% (*n* = 137) of all patients affected were women (*p* = 0,049). The median ages of all the patients were 84 and 86 years, respectively (*p* = 0,034).

The values of the ISAR score also differed (*p* = 0,035). The majority of patients had an ISAR score ≥ 2 (80.1% in the RA group vs. 84.5% in the ORIF group), indicating that increased geriatric measures are necessary.

The ability to walk independently was severely restricted seven days after surgical treatment. In total, 31.2% of the patients (*n* = 122) in the RA group and 45.5% (*n* = 80) in the ORIF group were immobile. This difference reached statistical significance (*p* = 0.001). Almost all patients were dependent on aids for mobilization. In the RA group, full weight bearing was permitted in 54.9% of patients; in the ORIF group, full weight bearing was permitted in only 37.0% of all patients (*p* < 0.001).

There was a significant difference in nonsurgical complications postoperatively (*p* = 0.003). Complications such as delirium, cystitis, pneumonia, renal insufficiency, decubitus, pulmonary embolism, thrombosis, and cardiac infarction occurred in 46.8% of the patients in the RA group but only in 33.5% of the patients in the ORIF group.

For a higher-quality comparison, 2:1 pair matching was performed for sex, age, ASA score and prefracture walking ability. A total of 528 patients were assigned according to nearest neighbor pair matching: 326 (66.7%) in the implant RA group and 163 (33.3%) in the ORIF group.

For this reason, the analysis of sex, age, ASA score and walking ability revealed no significant differences for Vancouver type B2 or B3 PFF. In the RA group, 69.6% (*n* = 227) and in the ORIF group, 73.6% (*n* = 120) of all the patients affected were women. The median age was 85 years in both groups (*p* = 0,266). Most patients were classified as ASA 3 in both groups (71.2% in both groups; *p* = 0,832). Almost all the patients were able to walk or at least had some ability to walk using walking sticks, crutches, or walkers. Only a minority of patients were bedridden before the acute fracture event (4.3% vs. 4.3%) (*p* = 0,900).

Prior to fracture, patients predominantly lived at home (88.6% in the RA group vs. 81.0% in the ORIF group; *p* = 0,051). The CFS score did not significantly differ between the two groups (*p* = 0,908). The values of the ISAR score also showed no significant differences (*p* = 0,062). The majority of patients had an ISAR score ≥ 2 (81.1% in the RA group vs. 84.7% in the ORIF group), indicating that increased geriatric measures were necessary.

In the RA group, 60.1% of patients received anticoagulation therapy at the time of the acute event compared to 58.6% in the ORIF group (*p* = 0,836). A total of 9.3% and 7.5% of patients had a concomitant injury, respectively (*p* = 0,614).

Positive delirium screening results were 18.8% and 17.9%, respectively (*p* = 0,959). The mortality rates during acute hospitalization were 6.8% and 4.3%, respectively (*p* = 0.379). The reoperation rates remained insignificant, with 10.6% of patients in the RA group and 5.3% in the ORIF group requiring reoperation during acute hospitalization (*p* = 0.089).

The ability to mobilize independently was severely restricted seven days after the surgical procedure. In total, 31.1% (*n* = 96) of the patients in the RA group and 44.5% (*n* = 69) in the ORIF group were immobile. This difference reached statistical significance (*p* = 0.006). Almost all patients were dependent on aids for mobilization. In the RA group, full weight bearing was permitted in 54.5% of patients, whereas in the ORIF group, weight bearing was allowed in only 36.9% of patients (*p* < 0.001).

Nonsurgical complications occurred significantly more often in the RA group than in the ORIF group (46.9% vs. 35.4%) (*p* = 0.023). Only 21.2% of patients in the ORIF group but 23.0% of patients in the RA group were discharged home (*p* = 0,038). The quality of life seven days after the operation, as measured with the EQ-5D-5 L, was not significantly different between the groups of patients (*p* = 0.866).

### 120-day follow-up data

For 150 patients, 120-day follow-up data were available. Among these patients, 104 (69.3%) were in the RA group, and 46 (30.7%) were in the ORIF group (Table 2). Unmatched data for these patients remained insignificant with regard to mortality, reoperation rate, place of residence, walking ability and EQ-5D-5 L score.

**Table 2:** 120 days follow-up data on patients with periprosthetic femoral fractures type Vancouver B2/B3

Parameter	All patients (before matching)			After matching		
	Revision arthroplasty (RA)	Osteosynthesis (ORIF)	*p*-value	Revision arthroplasty (RA)	Osteosynthesis (ORIF)	*p*-value
Number of patients	104 (69,3%)	46 (30,7%)		91 (68,9%)	41 (31,1%)	
Death within the first 120 days postoperative***						
Yes	9 (8,7%)	4 (8,7%)	1,000	8 (8,8%)	3 (7,3%)	1,000
No	95 (91,3%)	42 (91,3%)		83 (91,2%)	38 (92,7%)	
Reoperation within the first 120 days postoperative***						
Missings (before matching 13/after matching 0)			0,323			0,551
Yes	5 (5,3%)	0 (0,0%)		3 (3,6%)	0 (0,0%)	
No	90 (94,7%)	42 (100%)		80 (96,4%)	38 (100%)	
Residence at the time of the 120 days follow-up***						
At home	71 (68,3%)	27 (58,7%)	0,463	61 (67,0%)	25 (61,0%)	0,662
Geriatric rehabilitation-clinic	1 (1,0%)	0 (0,0%)		1 (1,1%)	0 (0,0%)	
Nursing home	21 (20,2%)	15 (32,6%)		19 (20,9%)	13 (31,7%)	
Hospital trauma unit	2 (1,9%)	0 (0,0%)		2 (2,2%)	0 (0,0%)	
Death	9 (8,7%)	4 (8,7%)		8 (8,8%)	3 (7,3%)	
Walking ability at the time of the 120 days follow-up***						
Missings (before matching 15/after matching 13)			0,738			0,461
Independent without walking aids	6 (6,4%)	5 (12,2%)		5 (6,1%)	5 (13,5%)	
Ability to walk inside	11 (11,7%)	4 (9,8%)		10 (12,2%)	4 (10,8%)	
Ability to walk with walking stick or crutch	17 (18,1%)	7 (17,1%)		14 (17,1%)	6 (16,2%)	
Ability to walk with two crutches or a walker	48 (51,1%)	22 (53,7%)		41 (50%)	20 (54,1%)	
No functional walking ability	12 (12,8%)	3 (7,3%)		12 (14,6%)	2 (5,4%)	
EQ-5D-5 L Index Median [IQR] *	0,779 [0,533, 0,888]	0,766 [0,414, 0,856]	0,358	0,758 [0,485, 0,884]	0,718 [0,390, 0,851]	0,472

* Wilcoxon-sign-rank test ** Pearson-Chi-square test. *** Exact test according to Fisher IQR: interquartile range

For further analysis, matched pairs were included in both groups. Patient data at the 120-day follow-up were available for 132 patients (91 patients (68.4%) in the RA group and 41 patients (31.1%) in the ORIF group) (Table 2). After matching, no significant differences were detected between the groups.

The mortality rate after 120 days was 8.8% in the RA group and 7.3% in the ORIF group (*p* = 1,000). The reoperation rate 120 days after the initial surgery was 3.6% in the RA group and 0.0% in the ORIF group (*p* = 0.551).

With rates of 67.0% in the RA group and 61.0% in the ORIF group, most patients lived at home at the time of data collection (*p* = 0,662).

Differences in walking ability remained insignificant. In the RA group, 14.6% of the patients were immobile, while only 5.4% were immobile in the ORIF group (*p* = 0,461). Only 6.1% and 13.5% were able to walk without aids after RA and ORIF, respectively. In addition, the quality of life measured according to the EQ-5D-5 L was not significantly different between the groups. The EQ-5D-5 L score was reduced to 0.758 and 0.718, respectively (*p* = 0,472).

### Multivariate analysis

After all patient data for age, sex, ASA score, and prefracture walking ability were analyzed, no differences were detected between the two treatment groups in terms of mortality during acute hospitalization, reoperation during acute hospitalization or quality of life as indicated by the EQ-5D-5 L score seven days after surgery (*p* = 0.736). Additionally, after 120 days, mortality and quality of life did not significantly differ between the two groups.

Multivariate logistic and linear regression analyses of RA vs. ORIF revealed significant differences for the parameter “ability to walk after seven days” (*p* = 0.007), for postoperative full weight bearing after RA (*p* < 0.001) and for nonsurgical complications after RA (*p* = 0.013).

Multivariate logistic and linear regression analyses of the parameters collected at the 120-day follow-up revealed that the OR for walking ability after seven days was significantly lower for patients treated with ORIF (OR: 0.55; *p* = 0.007). The OR for full weight bearing allowed was also significantly lower in this group (OR: 0.48; *p* < 0.001). The OR for the occurrence of nonsurgical complications was significantly lower in this group (OR: 0.6; *p* = 0.013) (Table 3).

**Table 3:** Multivariate logistic and linear regression analysis of RA vs. ORIF including all patients. Analysis is adjusted for age, gender, ASA score and walking ability before fracture

Parameter (osteosynthesis)	Number of observations	OR/β	95% lower CI	95% upper CI	*p*-value
Acute phase					
Mortality (yes)*	528	0,64	0,25	1,63	0,352
Walking ability after 7 days (yes)*	498	0,55	0,36	0,85	0,007
Reoperation during stay in the acute hospital (yes)*	501	0,47	0,21	1,06	0,068
Full weight bearing allowed (yes)*	519	0,48	0,32	0,71	< 0,001
Non-surgical complications (yes)*	515	0,6	0,4	0,9	0,013
Eq. 5D-5 L index after 7 days ~	392	0,01	−0,05	0,07	0,736
120 days follow-up					
Death during follow-up (yes)*	143	1,45	0,33	6,45	0,626
EQ-5D-5 L index after 120 days ~	121	0,01	−0,12	0,14	0,911

* logistic regression//~ linear regression

Similar results were observed in both groups after matching (Table 4). After controlling for age, sex, ASA score, and walking ability, no differences were detected between the RA group and the ORIF group in terms of mortality during acute hospitalization (*p* = 0.283), reoperation during acute hospitalization (*p* = 0.066) or quality of life as indicated by the EQ-5D-5 L score seven days after surgery (*p* = 0.990). Additionally, analysis of mortality after 120 days (*p* = 0.777) and the EQ-5D-5 L score (*p* = 0.546) revealed nonsignificant differences between the groups.

**Table 4:** Multivariate logistic and linear regression analysis of RA vs. ORIF after the matching. Analysis is adjusted for age, gender, ASA score and walking ability before fracture

Parameter (osteosynthesis)	Number of observations	OR/β	95% lower CI	95% upper CI	*p*-value
Acute phase					
Mortality (yes)*	489	0,62	0,26	1,48	0,283
Walking ability after 7 days (yes)*	464	0,56	0,38	0,84	0,005
Reoperation during stay in the acute hospital (yes)*	463	0,47	0,21	1,05	0,066
Full weight bearing allowed (yes)*	481	0,49	0,33	0,72	< 0,001
Non-surgical complications (yes)*	476	0,59	0,39	0,89	0,012
Eq. 5D-5 L index after 7 days ~	364	0,00	−0,07	0,07	0,99
120 days follow-up					
Death during follow-up (yes)*	132	0,82	0,21	3,26	0,777
EQ-5D-5 L index after 120 days ~	114	−0,04	−0,17	0,09	0,546

*Logistic regression//~ linear regression

Multivariate logistic and linear regression analyses of RA vs. ORIF after matching revealed significant differences in the ability to walk after seven days (*p* = 0.005), full weight bearing (*p* < 0.001) and nonsurgical complications (*p* = 0.012).

Multivariate logistic and linear regression analyses of the parameters collected at the 120-day follow-up revealed that the ORs for walking ability after seven days (OR: 0.56; *p* = 0.005), full weight bearing allowed (OR: 0.49; *p* < 0.001) and the occurrence of nonsurgical complications (OR: 0.59; *p* = 0.012) were significantly lower in the ORIF group.

## Discussion

The aim of the present evaluation of the ATR-DGU was to determine whether RA or ORIF is beneficial for the surgical treatment of Vancouver type B2 and B3 fractures in a geriatric patient population. On the basis of a 2:1 matched pair analysis, major study results indicated that patients treated with RA were significantly more often mobilized under full weight bearing postoperatively, leading to improved walking ability seven days after surgery. Nevertheless, the rate of nonsurgical complications increased significantly following RA, at least in the short term. In the midterm (120 days follow-up), all the examined parameters were comparable between the two treatment groups, especially in terms of quality of life, mortality, mobility and reoperation rate.

The results of the present analysis therefore confirm those of Chatziagorou et al.. who reported on data from the Swedish arthroplasty registry [23]. By analyzing the clinical courses of 801 patients treated for Vancouver type B2 or B3 fractures, they reported that revision with a longer femoral component resulted in a higher rate of immediate postoperative weight bearing than that in patients treated with plate fixation alone [23]. Similar results were published by *Gitajn et al.*, who reported on data from 121 patients treated for Vancouver type B2 or B3 PFF [24]. A retrospective review of patients at three tertiary referral academic medical centers in the USA revealed that their patients’ collective RA was significantly associated with increased rates of full weight bearing.

These results suggest that patients who are allowed to bear full weight show an improvement in their walking ability, even in the short term. In the present study, significantly better walking ability was detected in the RA group than in the ORIF group seven days after surgery. Although our results revealed inferior short-term functional outcomes following ORIF, *Moreta and coworkers* reported that in addition to good radiological results following cementless stem revision for Vancouver type B2 or B3 PFF, there was marked functional deterioration in many of their patients. None of their patients improved their ability to walk after these fractures, and more than half of them had not regained their prefracture walking status at the most recent follow-up ^25^. *Ueyama et al.* somewhat support these findings, as they reported their results on early postoperative functional recovery in older patients with PFF [26]. Having performed a retrospective cohort study on 18 patients with B2 PFF and a follow-up period of about two years, they could prove that patients receiving cemented stem revision showed faster recovery than those with uncemented stem revision. These improved functional abilities were evident in terms of a higher functional independence measure score, a higher independent walking rate and greater recovery in activities of daily living two weeks postoperatively [26].

In general, the age of patients with PFFs is high. The median age of our patients was 85 years, and women were far more likely to be affected by PFF than men were. Poor general health status, preexisting comorbidities, age over 65 years, male sex, neurological diseases, osteoporosis and heart failure are considered important risk factors for perioperative mortality and poor functional outcomes, not only for patients with hip fractures but also for those with PFFs [27–33].

Overall mortality was high in the present study (6.8% during hospitalization and 8.8% up to 120 days after surgery) and has been reported in the literature as high as 14.1% [13, 16, 34–36]. For this reason, efforts should be made to minimize the risk of complications. Most of the patients included in the present evaluation were classified as having an ISAR score ≥ 2 and therefore had an increased risk of health deterioration or functional limitations after discharge [37]. Additionally, the ASA score among the patients included was elevated in the present analysis, with most of the patients being classified as having an ASA score ≥ 3. In a systematic review and meta-analysis on surgical treatment for Vancouver type B2 PFFs, Gonzáles-Martín et al. reported that patients with an ASA score >3 underwent ORIF more frequently [38]. In this context, they emphasize the use of ORIF in selected patients with low functional demands, multiple comorbidities, and high anesthetic risk. A recently published retrospective study from Lampert et al. confirmed these findings. In their study with an age- and sex-matched study population, ORIF was performed among patients with higher ASA grades. In total, ORIF was associated with a shorter operation time and less blood loss [39].

If such a preselection is not performed, ORIF for Vancouver type B2 or B3 fractures may increase the revision rates and might be associated with higher complication rates, as Chatziagorou et al. and Khan et al. demonstrated [23, 40].

 Powell-Bown et al. compared PFF treated with ORIF to that with RA using Exeter stems (the most common stem in the UK; cemented, polished, and tapered). Their results revealed that patients who received ORIF had a lower risk of blood transfusion. With respect to complication rates and mortality rates, comparable results have been reported [20]. Slullitel and coworkers further support these findings, as they reported that low-demand, elderly patients with Vancouver type B2 fractures around well-cemented polished femoral components with an intact bone–cement interface can be safely treated with internal fixation [41]. In a further development of the Vancouver classification, Stoffel et al. named the fracture pattern, where an intact cement mantle remains, B2 ‘stable’ fracture [42]. According to data from the Swedish arthroplasty registry, these results may also be transferable to cementless stems [23].

The results of the present evaluation support the findings presented above. Additionally, in the present study, a significantly lower rate of nonsurgical complications was observed after ORIF. Avoiding such perioperative complications is crucial, not only in view of the potentially fatal outcomes but also in view of the impairment of quality of life. In recent literature, this point is becoming increasingly important, as not only survival but also postoperative living conditions have come to the fore. In this context, Pavlović and coworkers reported on health-related quality of life (EQ-5D) after RA following Vancouver type B2 and B3 PFFs in geriatric trauma patients. A retrospective analysis of 43 patients revealed a quality of life index of 0.8 ± 0.1, with more than 75% of surviving patients living at home one year postoperatively [13]. Additionally, Taha et al. reported on 25 patients who underwent ORIF for traumatic PFF in a university hospital in Egypt. In this study, the focus was on Vancouver type A, B1 or C fractures. Nevertheless, according to the EQ-5D-5 L assessed at the final follow-up, no patient felt that their daily life and activities had become more problematic [43]. These results in the current literature show that regaining preoperative quality of life seems possible after both RA and ORIF.

In our study, we also demonstrated that there were no differences between the RA group and the ORIF group in terms of quality of life as measured by the EQ-5D-5 L, after either seven or 120 days. There were also no significant differences with regard to the revision rate or mortality, which can also be confirmed in the literature. In a retrospective analysis of eleven centers in the USA, Toci et al. reported that patient age and fracture type (B3 versus B1) influenced reoperation rates. Treatment type had no influence on reoperation rates, and the influence of surgical training was unclear [19]. Additionally, Pohl et al. reported that there was no difference in terms of revision surgery, mobility or complications. Interestingly, they verified that the type of surgical training determined the choice of procedure for Vancouver type B fractures. Whereas trauma surgeons were significantly more likely to opt for ORIF, revision procedures were more common among arthroplasty surgeons [18].

### Limitations

Since the present analysis is based on registry data, several limitations must be considered. First, it should be noted that the analysis was conducted retrospectively, which may lead to potential biases such as confounding factors and selection bias. While well-designed randomized trials can prove causality, registry analyses, such as the present one, are only able to describe associations. Our findings must therefore be interpreted with caution. However, data for the ATR-DGU are collected prospectively, alleviating some of these concerns. Second, importantly, the validity of registry data depends crucially on accurate data collection and data entry. To ensure the integrity of these data, a certification process and regular audits are carried out for all hospitals participating in the ATR-DGU. Despite these limitations, the overall high number of participants included definitively strengthens the results of the present registry analysis. Furthermore, with the inclusion of patients from more than 150 geriatric trauma centers throughout Germany, Switzerland and Austria, the present study provides a comprehensive overview of the current treatment strategies and outcomes related to Vancouver type B2 and B3 PFFs in central Europe.

## Conclusions

RA has been considered the undisputed gold standard for the treatment of Vancouver type B2 and B3 PFFs. However, an increasing number of publications indicate that ORIF represents a good treatment alternative, provided that it enables a stable prosthesis fit with axial alignment.

**Fig. 1:**
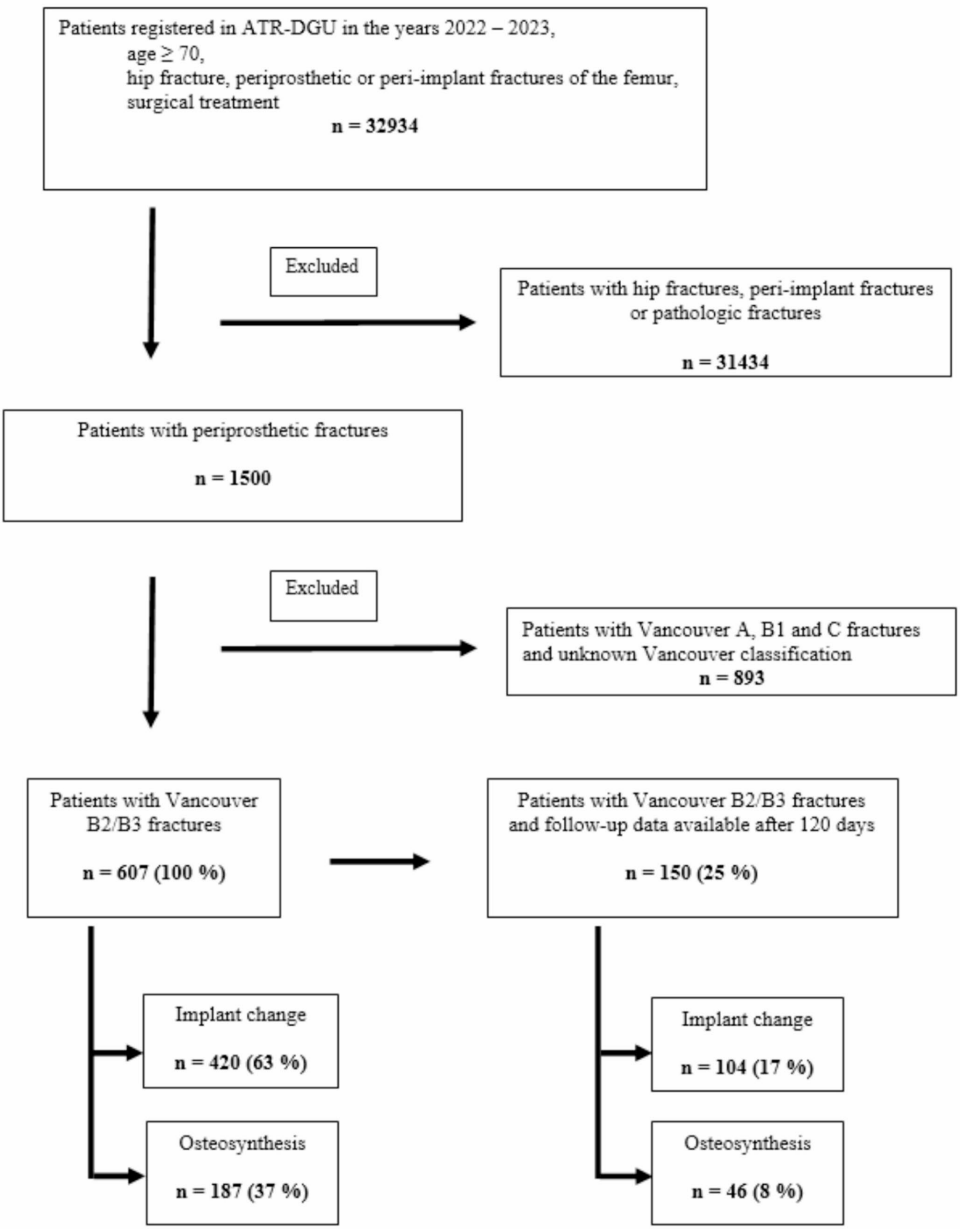
Flow sheet of the included population

The results of the present study support this point, as no relevant differences in the medium-term outcomes of the individual patient groups with regard to either treatment option were shown. Nevertheless, an individualized decision should always be made, especially in multimorbid geriatric patients, to optimize clinical courses and reduce complications (Fig. 1).

## Data Availability

No datasets were generated or analysed during the current study.
